# Metabolic trade-offs in tropical and subtropical marine mammals: unique maintenance and locomotion costs in West Indian manatees and Hawaiian monk seals

**DOI:** 10.1242/jeb.237628

**Published:** 2021-08-06

**Authors:** Jason S. John, Nicole M. Thometz, Katharine Boerner, Laura Denum, Traci L. Kendall, Beau P. Richter, Joseph C. Gaspard, Terrie M. Williams

**Affiliations:** 1 University of California Santa Cruz, Coastal Biology Building, 130 McAllister Way, Santa Cruz, CA 95060, USA; 2 University of San Francisco, 2130 Fulton Street, San Francisco, CA 94117, USA; 3 Mote Marine Laboratory & Aquarium, 1600 Ken Thompson Pkwy, Sarasota, FL 34236, USA; 4 Pittsburgh Zoo & PPG Aquarium, One Wild Place, Pittsburgh, PA 15206, USA

**Keywords:** Diving, Swimming, Energetic, *Trichechus manatus latirostris*, *Neomonachus schauinslandi*, Dive response

## Abstract

Unlike the majority of marine mammal species, Hawaiian monk seals (*Neomonachus schauinslandi*) and West Indian manatees (*Trichechus manatus latirostris*) reside exclusively in tropical or subtropical waters. Although potentially providing an energetic benefit through reduced maintenance and thermal costs, little is known about the cascading effects that may alter energy expenditure during activity, dive responses and overall energy budgets for these warm-water species. To examine this, we used open-flow respirometry to measure the energy expended during resting and swimming in both species. We found that the average resting metabolic rates (RMRs) for both the adult monk seal (753.8±26.1 kJ h^−1^, mean±s.e.m.) and manatees (887.7±19.5 kJ h^−1^) were lower than predicted for cold-water marine mammal species of similar body mass. Despite these relatively low RMRs, both total cost per stroke and total cost of transport (COT_TOT_) during submerged swimming were similar to predictions for comparably sized marine mammals (adult monk seal: cost per stroke=5.0±0.2 J kg^−1^ stroke^−1^, COT_TOT_=1.7±0.1 J kg^−1^ m^−1^; manatees: cost per stroke=2.0±0.4 J kg^−1^ stroke^−1^, COT_TOT_=0.87±0.17 J kg^−1^ m^−1^). These lower maintenance costs result in less variability in adjustable metabolic costs that occur during submergence for warm-water species. However, these reduced maintenance costs do not appear to confer an advantage in overall energetic costs during activity, potentially limiting the capacity of warm-water species to respond to anthropogenic or environmental threats that require increased energy expenditure.

## Abstract (precis)

**Summary:** An examination of energetics in manatees and monk seals provides insight into the metabolic trade-offs required for rest and diving by marine mammals living in warm water.

## INTRODUCTION

Thermoregulation and locomotion represent two of the most energetically costly physiological demands for marine mammals (Davis, 2014; Gallivan et al., 1983; Noren et al., 1999; Whittow, 1987; Williams et al., 1999). Maintaining homeothermy can be especially challenging because of elevated levels of heat transfer while in water (Whittow, 1987). To maintain thermal balance, many marine mammals exhibit higher maintenance metabolic rates than terrestrial mammals of similar body mass (Williams et al., 2001). This, in turn, leads to elevated food consumption rates, which necessitate increased investment in the daily energy expended for foraging activities (Rosen et al., 2007).

Theoretically, living in warm water should reduce these maintenance costs owing to a decrease in the thermal gradient for heat transfer compared with cold-water marine mammal species. In view of this, optimal energetic theory would predict an advantage for warm-water species. However, we find that nearly all marine mammal lineages, including those comprising the largest and smallest marine mammal species, exhibit increased species diversity in polar and temperate regions (Kaschner et al., 2011; Pompa et al., 2011). Of the 129 extant marine mammal species, less than 15% are found exclusively in subtropical or tropical waters (IUCN, 2020). These distributions are driven in part by the higher primary productivity, and hence food resource availability, of colder marine regions. Thus, the majority of marine mammal species appear to maintain energetic balance by taking advantage of this increased prey availability to compensate for the elevated maintenance demands associated with living in cold water. Superior insulation in the form of thick blubber layers, novel fur structures and densities, and modified dermal perfusion provide an additional thermal advantage, and have allowed marine mammals to radiate into some of the most thermally challenging habitats on Earth (Castellini, 2009; Whittow, 1987).

For the few marine mammal species that live in tropical regions, lower productivity and increased competition from ectotherms (Tittensor et al., 2010) as well as elevated anthropogenic impacts (Merchant et al., 2014; New et al., 2013; Nowacek et al., 2004) represent unique challenges to maintaining daily energy balance. Species in warmer habitats must also contend with overheating during physical exertion as a result of decreased perfusion of blood to the extremities and lower gradients for heat loss through the blubber layer, especially when diving (Noren et al., 1999; Pabst et al., 2002; Scholander et al., 1950). Previous studies have hypothesized that the avoidance of excess heat retention and consequent thermal imbalance occurring in warm-water habitats has necessitated the maintenance, and in some cases a reduction, in heat production for tropical marine mammals relative to polar or temperate congeners (Davis, 2019). This is evident in the lower basal metabolic rates (BMRs) and resting metabolic rates (RMRs) reported for some warm-water species (Davis, 2019). Such environmentally dependent adaptability (Lovegrove, 2005; Speakman, 1997) has obvious benefits for sustaining lower maintenance costs in resource-limited habitats. What is unclear is whether this low metabolic rate would also be accompanied by lower overall investment in locomotor costs relative to cold-water marine mammals.

As noted above, locomotion represents a significant component of the overall energy demands for animals, and defines an individual's ability to acquire food, avoid predation, as well as locate and move to suitable habitats (Maresh et al., 2015; Shepard et al., 2013; Williams et al., 2015, 2017a). In general, aquatic locomotion results in comparatively high transport costs in mammals, requiring significant morphological and physiological adaptations for oxygen conservation during swimming and diving (Feldkamp, 1987; Fish, 1994, 1998; Fish et al., 2008). Maintaining energy balance is further complicated in marine mammals by breath-holding and the dive response, which can alter the relationship between maintenance costs and locomotor costs as the animals surface and submerge (Brown et al., 2004; Wikelski and Cooke, 2006; Williams et al., 2006). Given the finite oxygen available to a marine mammal during a breath-hold dive, oxygen conservation is essential and requires efficiently balancing maintenance costs and locomotor costs to maximize activity while minimizing unnecessary oxygen use during the dive (Davis, 2019; Favilla and Costa, 2020; Ponganis, 2015). If the lower maintenance costs found in these warm-water species were accompanied by lower locomotor costs or resulted in lower total activity costs, it would result in a marked decrease in energetic expenditure during diving relative to cold-water marine mammal species.

In this study, we evaluated whether the energetic costs for maintenance and locomotion are altered with tropical or subtropical living by marine mammals, by measuring the metabolic responses of two warm-water species representing distinct evolutionary lineages: Hawaiian monk seals [*Neomonachus schauinslandi* (Matschie 1905)] and West Indian manatees [*Trichechus manatus latirostris* (Harlan 1824)]. Extant monk seal species are considered basal evolutionary forms of the phocid lineage (Scheel et al., 2014). They are found exclusively in the warm waters around Hawaii and the Mediterranean Sea, despite being closely related to both temperate and polar species such as northern elephant seals (*Mirounga angustirostris*) and Weddell seals (*Leptonychotes weddellii*), respectively. Extant sirenians are similarly found in warm waters and include some of the oldest marine mammal lineages; they are the only remaining herbivorous group.

The energetic costs for maintenance and locomotor activities of these tropical species were determined by measuring surface and submerged resting metabolic rates, submerged swimming metabolic rate, the total cost per stroke and total cost of transport (COT_TOT_). We also evaluated the effect of the dive response on energy expenditure, by comparing the energetics of continuous surface swimming with submerged swimming in West Indian manatees. These data were then used to examine the metabolic variability that enables marine mammals to balance maintenance and locomotion costs during a dive.

## MATERIALS AND METHODS

### Animals

We conducted resting and swimming trials with two adult male West Indian manatees (manatee 1: 34 years old, 545 kg; manatee 2: 31 years old, 819 kg) at the Mote Marine Laboratory and Aquarium (Sarasota, FL, USA) and one adult male and one juvenile male Hawaiian monk seal (12 years old, 198 kg; 3 years old, 97 kg) at the Long Marine Laboratory (Santa Cruz, CA, USA). All animals were long-term residents (>12 months) of their respective facilities. Trials took place in saltwater pools with depths of 1.5 to 3 m for the manatees and 3 m for the monk seals. Water temperature was maintained between 25.0 and 27.8°C, which is within the thermoneutral zone for both species. Data collection occurred during spring and summer to minimize any effect from air temperature variability. Manatees were fed multiple times daily with an herbivorous diet of romaine lettuce, kale, carrots, beets and apples. Hawaiian monk seals were fed a mixed fish diet. Both diets were calculated to meet daily maintenance requirements and were supplemented with multivitamins. Training for specific behaviors occurred for 6–12 months before data collection, using positive reinforcement and operant conditioning techniques. Because free-ranging manatees graze for up to 8 h each day (Bengston, 1983), the manatees in this study were fed throughout data collection to best approximate wild conditions and facilitate training and data collection. Manatees are also hind-gut fermenters, resulting in distribution of digestive costs over ≥5 days (Gallivan and Best, 1986). As a result, manatees do not exhibit an increased metabolic rate after feeding, which would influence measurements in other marine mammals (Costa and Kooyman, 1984; Gallivan and Best, 1986). Monk seals remained fasted throughout all data collection trials. All procedures were approved by the Mote Marine Laboratory and University of California Santa Cruz Institutional Care and Use Committees following National Institutes of Health guidelines, and conducted under Marine Mammal Permits through the US National Marine Fisheries Service Office of Protected Species and the US Fish and Wildlife Service (no. MA770191-5).

### Experimental design

**Fig. 1.**
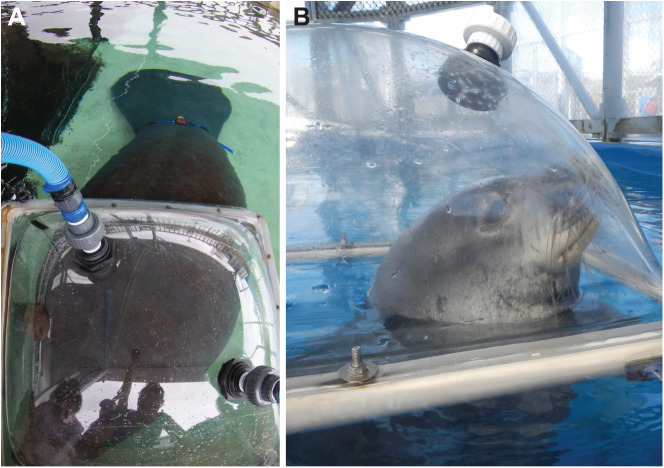
**Metabolic chambers for measuring oxygen consumption in West Indian manatees and Hawaiian monk seals.** (A) West Indian manatee; (B) Hawaiian monk seal. Resting and recovery behaviors were trained for 6–12 months prior to data collection to ensure quiescent behavior throughout trials.

**Fig. 2.**
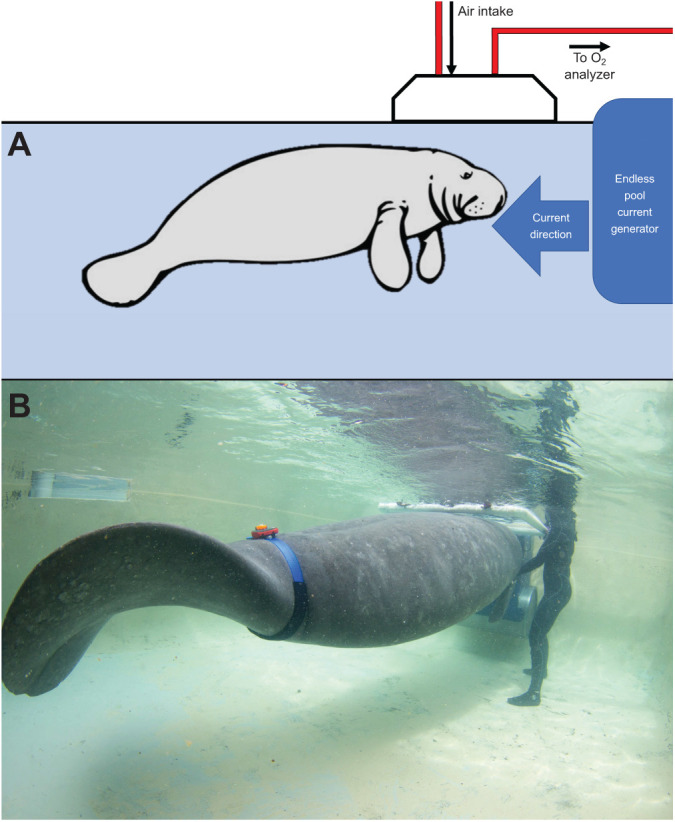
**Continuous surface swimming measured in West Indian Manatees.** (A) Swim flume and respirometry dome used for measuring the energetic cost of surface swimming in West Indian manatees. (B) Manatee 1 participating in swim measurement with accelerometer attached to a peduncle belt. Concurrent measurement of oxygen consumption and three-axis acceleration was performed for determination of both overall swim and individual stroke cost (photo credit Conor Goulding/Mote Marine Laboratory).

We used open-flow respirometry to measure oxygen consumption (*V̇*_O_2__) and evaluate energy expenditure. Oxygen consumption was measured using a plexiglass metabolic dome (manatee: 102×102×36 cm, monk seal: 160×100×60 cm) mounted on the water surface (Fig. 1). Stroke frequency was measured simultaneously during swims using acceleration recorded by animal-borne tags. Three experimental states were measured in both species: surface resting, submerged resting and submerged swimming. Surface swimming was also measured in manatees to evaluate the cost of transit swims commonly performed by this species in the wild (Fig. 2). During equipment set up prior to the start of data collection, animals stationed with a trainer while resting at the water surface for 10 to 15 min and were moved into position beside the metabolic dome 1–3 min immediately prior to data collection to prevent movement costs from influencing resting measurements. Data collection occurred during spring and summer to minimize effects from air temperature variability. Measurements represent average values taken over a minimum of 5 days (max 23 days) and during both spring and summer to prevent autocorrelation from measurements taken within a single day or season. Manatees participated in data collection no more than two times in a single day with a minimum of 3 h in between data collection periods. Monk seals were measured no more than once in a single day.

### Resting metabolic rate

For both species, RMR was determined while the animals stationed under a metabolic dome for 10–20 min while resting dorsal side up on the water surface with minimal movement. Monk seals (*n*=31 trials) were fasted throughout the measurement of RMR, and manatees (*n*=68 trials) were fed every 20–30 s during the measurements to simulate free-ranging conditions and maintain positioning.

### Submerged resting metabolic rate

Resting metabolic rate during submergence (RMR_sub_) was determined following dives to 3 m for durations of 2 to 8 min (adult monk seal: *n*=10 trials, juvenile monk seal: *n*=7 trials, manatee: *n*=20 trials). Animals held position dorsal side up on the bottom of the pool with minimal movement. Manatees were fed once every 30 s during submergence. Trials ended at the trainer's signal to surface inside the metabolic dome for measurement of oxygen consumption. As there is evidence for voluntary control of the dive response (McDonald et al., 2017; Ridgway et al., 1975), we were careful to avoid influencing data collection by maintaining open egress from the data collection area to ensure voluntary participation. As the animals were participating in dives of unknown length, however, it is possible this resulted in a larger reduction of metabolic rate than would otherwise be seen. Additionally, all submergence trials were within the calculated aerobic dive limit (cADL) based on an oxygen store of 20 ml O_2_ kg^−1^ for West Indian manatees (Davis, 2014) and 44.7 ml O_2_ kg^−1^ for Hawaiian monk seals (Thometz et al., 2015). After surfacing, the post-submergence metabolic rate was measured using the same behavioral protocols as for RMR.

### Energetic cost of submerged swimming

To measure the energetic cost of submerged swimming, the animals were trained to submerge to a depth of 1–2 m and remain submerged while swimming an 18 m circuit with continuous stroking until recalled to the dome by the trainer (adult monk seal: *n*=13 trials, juvenile monk seal: *n*=9 trials, manatee: *n*=14 trials). Swim speeds represented the preferred speeds for both manatees and monk seals and were performed for 1–2 and 4–6 min, respectively, to simulate typical dive durations (Reynolds, 1981; Wilson et al., 2017). Following each swim, the animals were signaled to return and surface inside a metabolic dome for measurement of recovery oxygen consumption as described above.

### Energetic cost of surface swimming

The energetic cost of surface swimming was measured in manatees (*n*=16 trials) using a continuous current generator (Endless Pools, Aston, PA, USA). The generator maintained current speeds of 0.3 to 0.5 m s^−1^ during data collection. Manatees were trained to station 15–30 cm in front of the current generator and maintain steady-state horizontal swimming for 5–15 min while surfacing inside a metabolic dome for breaths (Fig. 2). Food reinforcement was provided every 20–30 s as consistent with submerged trials. The metabolic dome was mounted on the water surface 10 cm in front of the current generator throughout data collection. Oxygen consumption was measured for 10–15 min prior to the start and after cessation of swimming to establish baseline resting levels for each trial.

### Data collection and analysis

#### Oxygen consumption

Oxygen consumption was measured with open-flow respirometry using protocols from Williams et al. (2004). Throughout surface rest and swimming and immediately following submerged trials, animals were trained to restrict their breathing to a plexiglass dome mounted on a PVC frame and resting on the water surface. Air was pulled through the dome at a rate of 250–400 l min^−1^ with a calibrated vacuum pump (FlowKit Mass Flow Generator, Sable Systems International Inc., North Las Vegas, NV, USA). Water temperature during data collection ranged from 25.0 to 27.8°C, and air temperature ranged from 15 to 36°C. The air flow rate was regulated and subsampled for oxygen content using a mass flow controller and oxygen analyzer (FoxBox Respirometry System, Sable Systems). Prior to oxygen analysis, subsamples were passed through a series of six tubes filled with desiccant (Drierite, W. A. Hammond Drierite, Xenia, OH, USA) and CO_2_ absorbent (Sodasorb, W. R. Grace & Co, Chicago, IL, USA). Subsample oxygen content was continuously monitored and recorded at 1 Hz using Expedata Analysis software (Sable Systems). These values were corrected for standard temperature and pressure and converted to *V̇*_O_2__ assuming a respiratory quotient of 0.76 for manatees (Ortiz et al., 1999) and 0.77 for monk seals (Davis et al., 1985), using equations from Withers (1977) and Fedak et al. (1981). The system was calibrated before each data collection period using dry ambient air (20.95% O_2_) and weekly with N_2_ gas according to the protocols of Fedak et al. (1981) and Davis et al. (1985).

For measurement of RMR, the animals stationed in the dome for 15–20 min with minimal movement. The lowest oxygen consumption measured for a minimum of 5 min for monk seals and 10 min for manatees was recorded for each trial. A baseline RMR was measured for submergence trials to calculate the metabolic rate during submergence and determine when the animal had fully recovered. The baseline RMR was measured prior to submergence for manatees and immediately following complete recovery from submergence trials for monk seals. Submerged resting and submerged swimming metabolic rates were measured by calculating the oxygen consumption during recovery that was in excess of RMR. When assessing the cost associated with stroking during a dive in manatees, extended stationary periods that occurred between the end of the swim and the first post-dive breath were subtracted from total dive costs. Stationary periods with no movement for longer than 5 s at the end of the dive were removed, assuming an oxygen consumption rate equal to submerged resting. Surface swimming metabolic rate in manatees was calculated as the average oxygen consumption measured throughout the swimming behavior after reaching a steady-state swim speed.

#### Swim mechanics

Swim mechanics were measured using submersible tri-axial accelerometers (manatee: CATS-Diary, Customized Animal Tracking Solutions, Oberstdorf, Germany; monk seal: HOBO Pendant G Data Logger, Onset Computer Corporation, Bourne, MA, USA). Acceleration was measured in m s^−2^ at 10 Hz for manatees and 20 Hz for monk seals and converted to ***g*** (1 ***g***=9.81 m s^−2^). For manatees, the accelerometer was attached on the dorsal center line at the peduncle using an aluminium mounting bracket attached to a nylon and neoprene strap. The frontal area of the CATS-Diary accelerometer and mounting bracket was 30 cm^2^ (<1% manatees frontal surface area). For monk seals, the accelerometer was attached around the left rear flipper using a neoprene strap. The frontal area of the HOBO accelerometer was 10 cm^2^ (<1% monk seal frontal surface area). Desensitization training started 6 months before data collection to prevent the attachment from influencing swimming mechanics. The total number of strokes per dive (*S*_dive_) was determined using *X*-axis acceleration (longitudinal axis) and counting a full stroke cycle as one individual stroke. *S*_dive_ was then divided by the total dive time in minutes to determine stroke frequency (*f_S_*, strokes min^−1^)*.* Because the animals were trained to swim continuously during diving, total cost per stroke (J kg^−1^ stroke^−1^) was analyzed, as determined by dividing the total energy expended during each dive (J kg^−1^) by *S*_dive_. Note that this differs from the net cost per stroke, often referred to as locomotor cost (Williams et al., 2004), in which maintenance costs are removed from the total oxygen consumption. Total cost of transport (COT_TOT_, J kg^−1^ m^−1^) was determined by dividing the total energy expended during the dive by the total distance the animal swam during the trial.

#### Analyses

Mixed-effects repeated-measures models were used to compare oxygen consumption rates during surface and submerged resting in all animals, as well as during surface and submerged swimming in manatees. Owing to the same individuals being sampled multiple times in both species, individual was treated as the subject in the repeated-measures approach. *P*<0.05 was used as the statistical threshold for significance in all tests. All analyses were conducted in R (https://www.r-project.org/) and JMP Pro (Version 14.3.0, SAS Institute Inc., Cary, NC, USA, 1989-2019). All results are presented as means±s.e.m. unless otherwise noted.

## RESULTS

### Resting metabolic rates

**Fig. 3.**
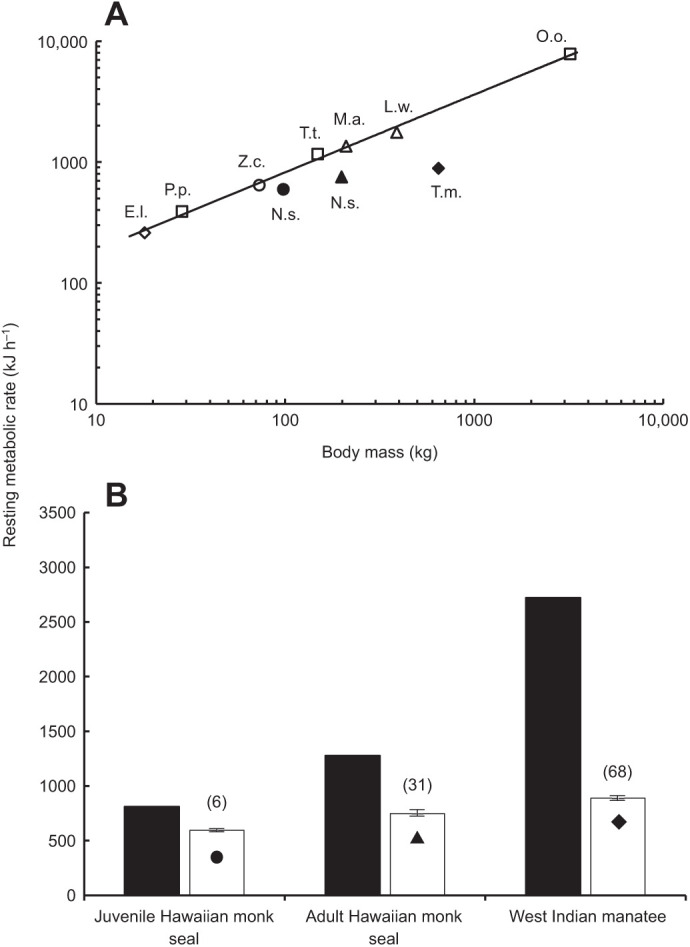
**Resting metabolic rate for marine mammals.** (A) Resting metabolic rate (RMR, kJ h^−1^) versus body mass (kg) for marine mammals. Solid line is the allometric regression for marine mammals stationing on the water surface adapted from Williams et al. (2001) (RMR=41.5 Mass^0.65^). Closed symbols represent mean RMR for the juvenile Hawaiian monk seal (N.s. closed circle), adult Hawaiian monk seal (N.s. closed triangle) and West Indian manatees (T.m. closed diamond). Open symbols represent mean RMR for sea otters (E.l. diamond; Williams, 1989), harbor porpoise (P.p. square; Kanwisher and Sundnes, 1965), California sea lions (Z.c. circle; Liao, 1990), bottlenose dolphins (T.t. square; Williams et al., 2001), northern elephant seals (M.a. triangle; Costa et al., 1986), Weddell seals (L.w. triangle; Castellini et al., 1992) and killer whales (O.o. square; Kriete, 1995). (B) Predicted (black bar, Williams et al., 2001) and measured (white bar) RMR (kJ h^−1^) for Hawaiian monk seals and West Indian manatees. Height of bar and lines represent mean±1 s.e.m. RMR. Mean RMR for West Indian manatees was 68% lower than predicted for similarly sized marine mammals, while RMR for the adult Hawaiian monk seal was 41% lower than predicted. RMR for the juvenile Hawaiian monk seal (Williams et al., 2011) was 27% lower than predicted.

The average RMR for the adult monk seal was 753.8±26.1 kJ h^−1^ (*n*=31). For comparison, the previously published average RMR for the juvenile monk seal (Williams et al., 2011) was 594.3±8.2 kJ h^−1^. The average RMR for both manatees combined was 887.7±19.5 kJ h^−1^ (*n*=68). RMR for manatee 1 was 945.6±27.8 kJ h^−1^ (*n*=35), and for manatee 2 was 826.4±23.4 kJ h^−1^ (*n*=33). Although the adult and juvenile monk seal values were 33% and 79% higher, respectively, than predicted for terrestrial carnivorous mammals as described by the equation RMR (kJ h^−1^)=0.06 Mass(g)^0.752^ (Davis, 2019), the RMR of the adult monk seal was 41% lower than predicted for a similarly sized marine mammal as described by the equation RMR (kJ h^−1^)=41.5 Mass(kg)^0.65^ (Williams et al., 2001). In comparison, the previously published value for the juvenile monk seal was 27% lower than predicted for marine mammals. RMR for both manatees combined was 68% lower than predicted for marine mammals in addition to being 63% lower than predicted for terrestrial carnivorous mammals (Fig. 3).

### Submerged resting metabolic rates

**Fig. 4.**
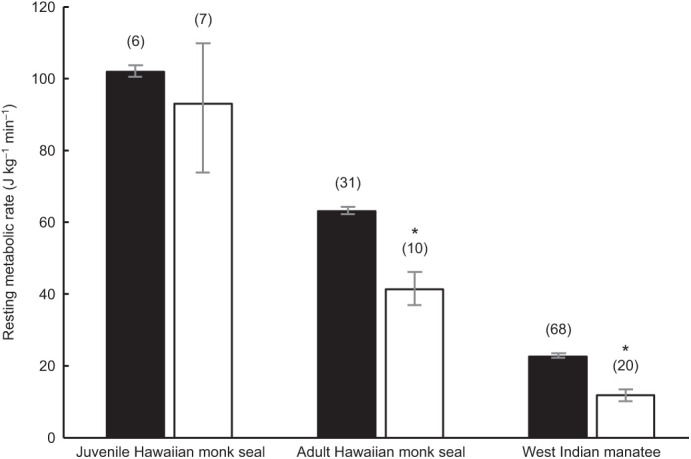
**Mass-specific RMR for tropical and subtropical marine mammals floating on the water surface (black bars) and submerged to 3 m (white bars).** Height of bar and lines represent mean±1 s.e.m. RMR. * indicates significant decreases in submerged values that were found in both the adult Hawaiian monk seal (35% decrease, *P*<0.0001, *n*=10 dives) and West Indian manatees (48% decrease, *P*<0.0001, *n*=20 dives). The juvenile Hawaiian monk seal exhibited a small but non-significant decrease from surface to submerged RMR (8% decrease, *n*=7 dives).

We found significant differences between submerged and surface RMR for both species (Fig. 4). The adult monk seal exhibited a RMR_sub_ of 41.3±4.2 J kg^−1^ min^−1^ (*n*=10), a 35% decrease from mass-specific RMR (63.1±2.4 J kg^−1^ min^−1^, d.f.=1,39, *F*=20.06, *P*<0.0001) while resting on the surface. The juvenile monk seal showed a non-significant decrease of 8% from 101.9 J kg^−1^ min^−1^ (RMR) to 93.0±15.1 J kg^−1^ min^−1^ (RMR_sub_, *n*=7). Average manatee RMR_sub_ was 11.8±1.3 J kg^−1^ min^−1^ (*n*=20), a 48% decrease from mass-specific RMR (22.6±0.6 J kg^−1^ min^−1^, d.f.=1,85, *F*=137.65, *P*<0.0001) while resting on the water surface. Manatee 1 exhibited an RMR_sub_ of 7.6±1.4 J kg^−1^ min^−1^ (*n*=10), a 61% decrease from mass-specific RMR (19.3±0.6 J kg^−1^ min^−1^, d.f.=1,41, *F*=57.05, *P*<0.0001). Manatee 2 exhibited an RMR_sub_ of 16.0±1.2 J kg^−1^ min^−1^ (*n*=10), a 39% decrease from mass-specific RMR (26.2±0.7 J kg^−1^ min^−1^, d.f.=1,43, *F*=81.38, *P*<0.0001) (Fig. 4).

### Energetic cost of submerged swimming

**Fig. 5.**
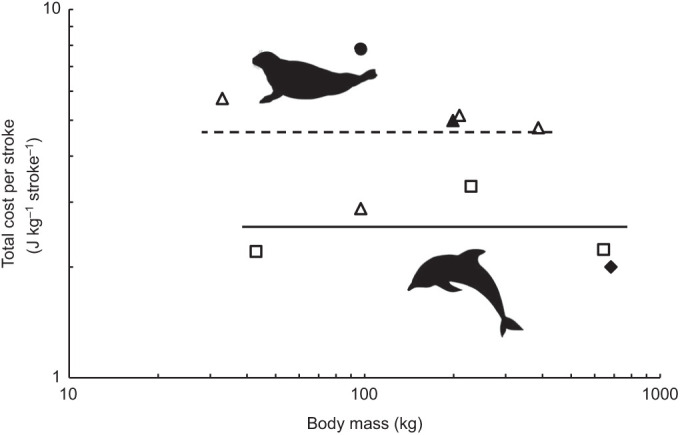
**Total cost per stroke (J** **kg** **stroke^−1^) versus body mass (kg) for swimming juvenile Hawaiian monk seal (closed circle), adult Hawaiian monk seal (closed triangle) and West Indian manatees (closed diamond) from the present study, in relation to swimming phocid seals (open triangles) and cetaceans (open squares).** Phocid and cetacean points represent mean stroke costs of harbor seals (Davis et al., 1985), harp seals (Fish et al., 1988; Innes, 1984), elephant seals (Maresh et al., 2014), Weddell seals (Williams et al., 2004), harbor porpoises (Otani et al., 2001), bottlenose dolphins and beluga whales (Williams et al., 2017b). Total cost per stroke is reported as the average cost per one full stroke cycle and calculated as described in the Materials and Methods. Lines represent the mean cost per stroke for phocid seals (dashed) and cetaceans (solid).

At an average preferred swimming speed of 1.4 m s^−1^ (range: 0.9 to 2.0 m s^−1^) measured in this study, the energetic cost of submerged swimming for the adult monk seal was 151.7±7.6 J kg^−1^ min^−1^ (*n*=13 dives), and for the juvenile monk seal was 229.5±23.5 J kg^−1^ min^−1^ (*n*=11 dives). The average total cost per stroke for the adult monk seal was 5.0±0.2 J kg^−1^ stroke^−1^ (*n*=13 dives), and 7.8±0.5 J kg^−1^ stroke^−1^ (*n*=9 dives) for the juvenile monk seal. These values are comparable to those reported by Williams et al. (2004) for Weddell seals (mean: 4.78 J kg^−1^ stroke^−1^) and by Davis et al. (1985) for harbor seals (mean: 5.74 J kg^−1^ stroke^−1^) and as predicted for stroke costs for other phocid seals (Fig. 5).

At the average preferred swimming speed of 0.34 m s^−1^ (*n*=14 dives, range: 0.26–0.41 m s^−1^), the energetic cost of submerged swimming for both manatees combined was 18.8±4.1 J kg^−1^ min^−1^. The average total cost per stroke was 2.0±0.4 J kg^−1^ stroke^−1^ (*n*=14 dives) (Fig. 5), which more closely compares with the stroke costs of cetaceans such as harbor porpoises (2.20 J kg^−1^ stroke^−1^; Otani et al., 2001) and bottlenose dolphins (3.31 J kg^−1^ stroke^−1^; Williams et al., 2017b).

### Total cost of transport

**Fig. 6.**
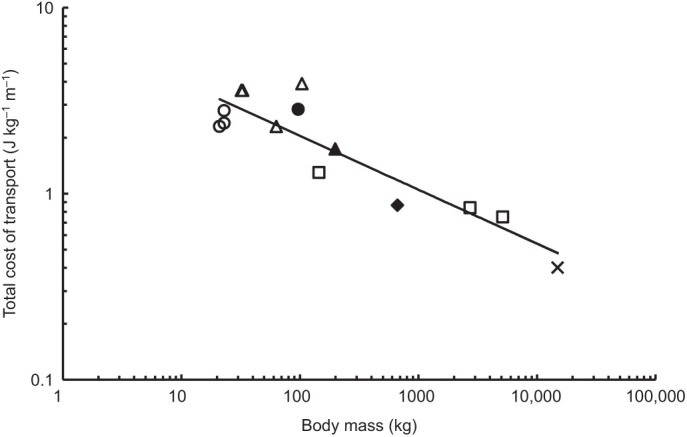
**Total cost of transport in relation to body mass for Hawaiian monk seals and West Indian manatees in relation to other swimming marine mammals.** Data for phocid seals (open triangles), California sea lions (open circles), bottlenose dolphins and killer whales (open squares), and gray whales (X) adapted from Williams (1999). The adult Hawaiian monk seal (closed triangle, 4% above predicted), juvenile Hawaiian monk seal (closed circle, 37% above predicted) and West Indian manatees (closed diamond, 26% below predicted) measured in this study exhibited an average COT_TOT_ similar to other swimming marine mammals despite significantly lower RMRs.

Despite measured adult RMRs that were 41% and 68% lower than predicted for the monk seal and manatee, respectively, COT_TOT_ values for adults of both species were within 26% of the predicted values for marine mammals as described by the equation COT_TOT_=7.79 Mass^−0.29^ (Williams, 1999) (Fig. 6). Average COT_TOT_ for the adult monk seal was 1.7±0.1 J kg^−1^ m^−1^ (*n*=12, 4% higher than predicted), and average COT_TOT_ for the juvenile monk seal was 2.8±0.4 J kg^−1^ m^−1^ (*n*=10, 37% higher than predicted). Average COT_TOT_ for both manatees combined was 0.87±0.17 J kg^−1^ m^−1^ (*n*=14, 26% lower than predicted).

### Energetic cost of surface swimming in manatees

**Fig. 7.**
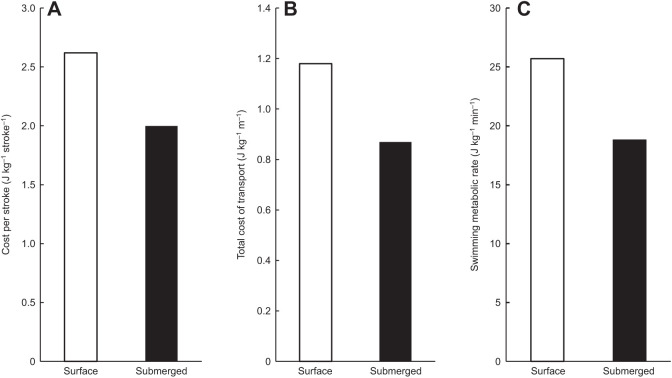
**Manatee surface and submerged energetic costs.** (A) Cost per stroke (J kg stroke^−1^), (B) total cost of transport (J kg m^−1^) and (C) swimming metabolic rate (J kg min^−1^) at the surface (white bars) and while submerged (black bars). Manatees exhibited similar relative decreases in metabolic rate in all three submerged metrics.

At an average sustained speed of 0.38 m s^−1^ (*n*=16 swims, range: 0.28–0.44 m s^−1^), the average energetic cost of surface swimming for the manatees was 25.7±1.7 J kg^−1^ min^−1^. This was a non-significant increase of 6.9 J kg^−1^ min^−1^ or 27% over the submerged swim cost (*n*=30, d.f.=1,27, *F*=3.63, *P*=0.0675). Average COT_TOT_ and average total cost per stroke also showed non-significant increases over submerged swim costs. Average COT_TOT_ increased by 27% to 1.18±0.1 J kg^−1^ m^−1^ (*n*=30, d.f.=1,27, *F*=4.00, *P*=0.0558) during surface swimming. The average total cost per stroke increased by 24% to 2.62 J kg^−1^ stroke^−1^ (*n*=30, d.f.=1,27, *F*=2.94, *P*=0.0980). Despite the limitations presented by a small sample size for comparison, these results indicate a distinct, though non-significant, difference in the energetic costs of surface and submerged swimming (Fig. 7).

## DISCUSSION

### The effects of tropical or subtropical living on energetic costs

In this study, we found that two independent lineages of marine mammals living in warm waters showed similar patterns in metabolic responses, that is, lower RMRs relative to similarly sized marine mammal species residing in colder habitats (Fig. 3). This might be expected, as the thermal gradient for tropical or subtropical species in warm water is lower than for temperate or polar relatives residing in cold water. For example, the mass-specific RMR of Hawaiian monk seals is 8% lower than the RMR measured for the closely related Antarctic Weddell seals (Castellini et al., 1992), despite the Hawaiian monk seal's smaller body size.

In the case of extant sirenians, the warmer environment and consequent lower metabolic demands can be supported by an herbivorous diet that is absent in all other marine mammal species. This diet, consumed in a continuous grazing mode of feeding, is sufficient for meeting the manatee's energetic demands that are extraordinarily low, even relative to the carnivorous monk seal. Although the increased bone density of sirenian species (Domning and de Buffrenil, 1991; Ingle and Porter, 2020) may partially account for the relative decrease in mass-specific RMR compared with other marine mammals (Rea and Costa, 1992), the effect is minimal compared with the combined effects of diet and environmental temperature (Costa and Maresh, 2017). Interestingly, the extinct Steller's sea cow also maintained an herbivorous diet but resided in the frigid waters of the Bering Sea. The large size of the sea cow likely contributed to this, as its estimated mass was 10 metric tons (Scheffer, 1972), or over 12 times the mass of the largest manatee in this study. The result would have been a thermally favorable relationship between surface area and volume for heat retention for the Steller's sea cow compared with the species' extant relatives. Assuming this translated to a decreased metabolic demand for heat production compared with other cold-water marine mammals, a plant-based diet appeared sufficient for sustaining this unusual Northern Pacific mammal.

Taken together, these findings also provide insight into how metabolic rates may have changed in marine mammals across evolutionary time. The origins of sirenians (Domning, 1982) and *Monachus* seals (de Muizon, 1982; Fyler et al., 2005) from the warm waters (23.5–36°C) of the Tethys Sea (Alsenz et al., 2013) would have facilitated a comparatively modest thermal transition from terrestrial to marine living for these mammalian lineages. Ultimately, the West Indian manatee has evolved one of the lowest relative resting metabolic rates for a mammal, even compared with the phylogenetically related terrestrial artiodactyls representing their original ancestry (Davis, 2019).

An alternative path has been proposed for Hawaiian monk seals in which the seals originated in the North Pacific along with many other pinniped species (Fyler et al., 2005; Hafed et al., 2020). In the case of the monk seal, a reduction in metabolic rate consequent with the return to tropical waters would have provided an adaptive benefit in the face of reduced prey availability typically found in warm marine environments (Villegas-Amtmann et al., 2017). Unlike the manatee, the Hawaiian monk seal exhibits a modest reduction in maintenance costs below similar cold-adapted marine mammals (Fig. 3) but close to what would be predicted for terrestrial carnivorous mammals (Kleiber, 1975) that are representative of the original ancestral physiological state.

Based on these metabolic patterns for tropical and cold-water species, a comparatively low RMR may represent the basal condition for marine mammals. This would have conferred an evolutionary thermal and energetic advantage, and thus be retained in those lineages that have continued to live in tropical waters (Berta et al., 2006). In comparison, species such as the Weddell seal and northern elephant seal that radiated to and remained in colder waters in pursuit of increased prey resources, have incurred higher total maintenance costs in response to an increased thermoregulatory load, and thus exhibit a higher RMR.

### Interrelationships between resting and active metabolic rates

Despite lower maintenance costs for warm-water marine mammals, we did not find a concomitant decrease in total locomotor costs or COT_TOT_ for the tropical marine mammals in this study. We attribute this to differences in the downregulation of maintenance costs that occur for tropical and cold-water marine mammals during submergence (Fig. 4). For example, the Hawaiian monk seal's RMR is approximately 41% lower than predicted for similarly sized marine mammals (Fig. 3), while the phylogenetically related Weddell seal has an RMR that is as predicted for a similarly sized marine mammal. However, despite these differences in RMR, both species exhibit similar costs per stroke, and COT_TOT_ for the monk seal was as predicted for similarly sized marine mammals. This unexpected similarity in total locomotor costs may be explained by differences in metabolic suppression that occur during submergence for the seals.

**Fig. 8.**
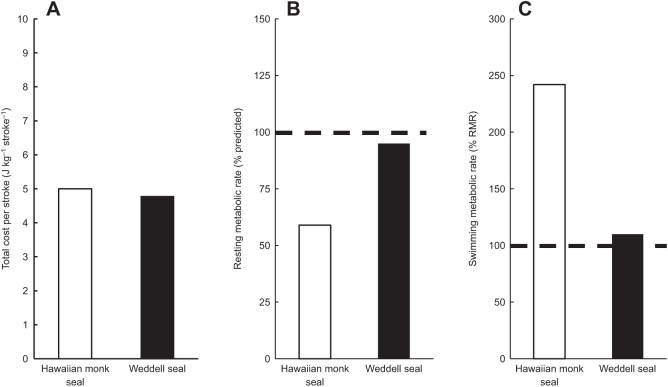
**Comparative energetic costs for Hawaiian monk seal and Weddell seals.** (A) Total cost per stroke (J kg stroke^−1^), (B) RMR relative to predicted value for a similarly sized marine mammal and (C) swimming metabolic rate relative to RMR for Hawaiian monk seal (white bar) and Weddell seals (black bar, Castellini et al., 1992; Williams et al., 2004). Dashed lines in B and C represent 100% predicted RMR and 100% actual RMR, respectively. Both species exhibit similar costs per stroke despite markedly different relative RMRs. Increased relative swimming metabolic rate in the Hawaiian monk seal over Weddell seals is a result of lower variable metabolic costs in Hawaiian monk seals. These decreased costs result in a lower RMR, but also decreased metabolic flexibility during a dive as there are fewer non-essential metabolic costs that can be downregulated by the dive response. This results in a higher relative and actual swimming metabolic rate when compared to Weddell seals.

**Fig. 9.**
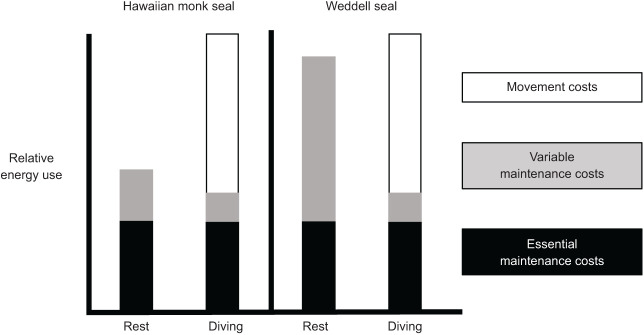
**Schematic representation of the relative energy used by Hawaiian monk seals and Weddell seals during rest and diving.** The lower energetic investment in variable maintenance costs such as thermoregulation during rest results in a lower mass-specific RMR for Hawaiian monk seals relative to Weddell seals. Conversely, the ability to downregulate those variable maintenance costs while submerged results in similar total locomotor costs for both species during diving.

By examining species-specific effects of submergence on the energetic cost of swimming, we can begin to fully understand why lower RMRs in tropical species do not confer a selective advantage in terms of the total energy expended during diving. In Hawaiian monk seals, the submerged swimming metabolic rate is approximately 2.4 times higher than its RMR (Fig. 8). In contrast, the Weddell seal's submerged swimming metabolic rate (90.45 J kg^−1^ min^−1^; Castellini et al., 1992) is only 1.1 times its RMR (Fig. 8). This marked difference is due to a limit in metabolic variability and trade-offs in metabolic compartments during underwater activity for warm-adapted and cold-adapted species as detailed below (Fig. 9).

In manatees, we were able to assess the cost of surface and submerged swimming to directly examine the effects of submergence on activity. These swimming modes are commonly used for cruising swims at the surface and submerged feeding dives, respectively (Reynolds, 1981). Both behaviors were performed within 1–2 m (<2.5 times body diameter) of the water surface, maintaining similar resistance from surface drag in both swimming modes (Williams, 1989). By matching activity levels for each mode, we found a distinct (although statistically non-significant) difference in the costs associated with these different locomotor modes (Fig. 7). Total swimming costs, total cost per stroke and COT_TOT_ were lower during submergence for the manatees, consistent with the downregulation of overall maintenance costs in association with the dive response as was seen in comparing surface and submerged resting costs.

Breaking down the energy budgets of each species into three compartments – essential maintenance costs, movement costs and variable maintenance costs – demonstrates how species-specific variability in each component contributes to the overall energy budget of these animals (Fig. 9). Essential maintenance costs describe the energy required for functions that continuously operate to ensure survival. These include maintaining blood flow to the heart and brain, basic cellular respiration, and postural muscle function, as well as growth and maturation in juveniles or fetal development during pregnancy. These costs are a product of basic biological necessity and thus determined primarily by evolutionary lineage and size (Schmidt-Nielsen, 1984). Movement costs include the energy expended for locomotor movements such as muscle contraction. As shown above, movement costs are also consistent across evolutionary lineages and determined primarily by muscle function, hydrodynamics and biomechanics (Williams, 1999; Williams et al., 2004).

Variable maintenance costs, as the name implies, refer to functions such as heat production and digestion that take place as needed throughout the body. Unlike essential maintenance costs or movement costs, variable maintenance costs are reactive to both the environment and the activities the animal undertakes, such as diving. These costs can increase to compensate for physiological needs such as restoration of oxygen stores, exercise recovery, acute thermoregulatory responses and tissue repair, or even responses to chronic disturbance (Holt et al., 2015; Williams et al., 2017a). Importantly, variable maintenance costs can also be downregulated when necessary to ensure metabolic fuel is conserved for essential maintenance costs particularly during diving (Favilla and Costa, 2020; Hill et al., 1987; Ponganis, 2015). This downregulation can occur on several scales, from long-term responses associated with low food resource availability or quality (Rea and Costa, 1992; Rosen and Trites, 1999) to short-term responses as occurs during prolonged diving (Davis, 2019; Davis and Williams, 2012).

As demonstrated by the differences in surface and submerged resting metabolic rates, and also indicated by the manatees during submerged versus surface swimming, metabolic downregulation instigated by the dive response when submerged serves to reduce variable maintenance costs. In this way, limited on-board oxygen is conserved (Davis, 2019; Ponganis, 2015). Vasoconstriction, which restricts blood flow to the core and essential tissues, and the deferment of digestion until after the dive represent some of the non-essential physiological processes that may be downregulated or delayed during submergence (Davis, 2014; Hindle et al., 2019; Noren et al., 1999; Zapol et al., 1979). As discussed by Favilla and Costa (2020), downregulating variable costs, including thermoregulation and digestion, helps to maximize efficiency during a dive by minimizing conflicting demands on limited oxygen stores. The combination of this downregulation of non-essential processes and increased heat production owing to muscle activity theoretically reduces the need for energy investment in variable maintenance costs while diving, particularly for polar species such as Weddell seals.

Species such as the Hawaiian monk seal, residing in warmer environments, incur lower overall maintenance costs while resting as a product of the reduced need for variable maintenance costs such as thermoregulation. Although beneficial during rest, these already low maintenance costs have a limited range for additional downregulation during a dive. The result is a limited capacity for reducing total swimming costs when movement costs are also included. Conversely, for polar species such as Weddell seals, the increased need for variable maintenance costs associated with thermoregulation at the surface can be significantly decreased during a dive. As a result, while surface costs are substantially affected by environmental temperature, total submerged swimming costs for these closely related phocid species appear to be independent of environmental temperature per se, and instead are driven by oxygen conservation. This reduces the cost of swimming relative to RMR in cold-water species and explains the lack of energetic advantage conferred by the low RMR in tropical or subtropical species. In manatees, this is demonstrated by the cost of swimming at the surface compared with both resting and submerged swimming. Because oxygen is not a limited resource while swimming at the surface, variable costs remain elevated and surface swimming incurs a higher total cost than either resting or swimming while submerged.

Further evidence for the balance between metabolic downregulation and swimming costs is provided by the juvenile Hawaiian monk seal measured in this study. Along with an elevated RMR compared with the adult conspecific, the juvenile monk seal also exhibited a high cost per stroke and COT_TOT_ relative to similarly sized adult marine mammals (Figs 5 and 6). In view of the increased essential maintenance costs associated with growth and maturation in the immature seal, costs that cannot be deferred during a dive, the measured increase in total locomotor costs relative to both the adult monk seal and other marine mammal species would be expected.

### Conclusions

This study has focused on marine mammal species from two unique lineages that inhabit tropical and subtropical waters. Clearly, the metabolic physiology of these animals differs from that of cold-water species. The latter appear to exhibit increased metabolic variability during a dive owing to increased reliance on variable maintenance costs that can be downregulated when submerged. The tropical and subtropical species studied exhibit a lower RMR overall as a result of decreased variable maintenance costs. Although this reduced metabolic variability, and thus did not translate to an energetic advantage during diving, the low RMR might still prove beneficial during recovery at the water surface. Conversely, the lower RMR could also manifest in a lower maximum aerobic scope, although this has yet to be investigated for warm-water marine mammals. This, combined with the elevated locomotor costs when fleeing threats, could potentially limit the energetic capacity of these species to respond to anthropogenic or environmental threats requiring increased energy expenditure. Further examination of the post-dive recovery energetics of these species would help to clarify this metabolic interaction.
